# Evaluating Bioactive-Substance-Based Interventions for Adults with MASLD: Results from a Systematic Scoping Review

**DOI:** 10.3390/nu17030453

**Published:** 2025-01-26

**Authors:** Deepa Handu, Kim Stote, Tami Piemonte

**Affiliations:** 1 Academy of Nutrition and Dietetics, Chicago, IL 60606, USA; tpiemonte@earthlink.net; 2 Department of Allied Health Sciences, State University of New York, Empire State University, Saratoga Springs, NY 12866, USA; kim.stote@sunyempire.edu

**Keywords:** MASLD, non-alcoholic fatty liver disease, bioactive substances, lifestyle management, scoping review

## Abstract

**Objective**: Metabolic dysfunction-associated steatotic liver disease (MASLD) is a chronic condition affecting a broad population. This review aimed to identify and summarize the current evidence on bioactive-substance-based interventions for adults with MASLD, formerly known as nonalcoholic fatty liver disease (NAFLD), covering publications from 2000 to 2023. **Methods**: A search was conducted across six databases (MEDLINE, CINAHL, Cochrane CENTRAL, Cochrane Database of Systematic Reviews, Food Science Source, and SPORTDiscus) for randomized controlled trials and other study types (e.g., prospective cohort studies and systematic reviews), reflecting the scoping nature of this review. The search was limited to studies in adults (>18 years old), with an intervention of interest and at least one comparator group. **Results**: A total of 4572 articles were retrieved, with 201 full-text articles screened for eligibility. Of these, 131 primary studies and 49 systematic reviews were included in the scoping review. The most studied bioactive substances were Curcumin (Turmeric) (n = 25), Silymarin (Milk Thistle) (n = 17), Resveratrol (n = 10), Coffee (n = 7), Green Tea (n = 5), and Berberine (n = 5 each). Moreover, 46 studies reported on 36 other bioactive substances with 2 or fewer articles each. Among the included systematic reviews, 13 focused on Curcumin, 12 on Coffee or Tea, 10 on bioactive substance combinations, 6 on Resveratrol, and 2 each on Silymarin and Artichoke Leaf. The included studies showed substantial heterogeneity in reported outcomes, which primarily focused on hepatic health, body weight, adverse events, glycemic control, blood lipids, and body composition. **Conclusions**: This scoping review highlights a range of bioactive substances used in the treatment of MASLD. While evidence is abundant for bioactive substances like Curcumin and Silymarin, further research and synthesis of findings is necessary to establish the clinical efficacy of all bioactive substances.

## 1. Introduction

Currently, few pharmacological treatments exist for metabolic dysfunction-associated steatosis liver disease (MASLD, previously NAFLD) or MASH, making lifestyle interventions such as weight loss and increased physical activity the cornerstone of management [1]. Thus, there is a growing public interest in dietary bioactive substances for hepatic health, which includes MASLD. The working definition for bioactives of the US National Institutes of Health Office of Dietary Supplements is “constituents in food or dietary supplements, other than those needed to meet basic human nutritional needs, that are responsible for changes in health status”. In addition, there is a framework for developing recommended intakes of dietary bioactive substances [2]. Several examples of dietary bioactive substances of public interest for MASLD are Curcumin (Turmeric), Silymarin (Milk Thistle), Resveratrol, and Coffee and Green Tea polyphenols. The US National Institute of Diabetes and Digestive and Kidney Diseases (NIDDK) estimates that up to 40% of patients attending hepatology clinics use some form of dietary bioactive substances [3]. Therefore, the objective of this Evidence Analysis Center scoping review is to identify and characterize the current evidence on dietary bioactive-substance-based interventions for adults, 18 years of age or older, with MASLD/NAFLD. This scoping review will inform research and future development of randomized controlled trials (RCTs) and systematic reviews (SRs) in this area.

## 2. Methods

This scoping review is based on the protocol developed by Arksey and O’Malley [4] and updated by Levac et al. [5] and the Joanna Briggs Institute [6]. The protocol for this project adheres to the Preferred Reporting Items for Systematic Reviews and Meta-Analyses checklist [7] for scoping reviews and has been registered on Open Science Framework [8]. Furthermore, this review was conducted under the guidance of a content advisor with expertise in MASLD and nutrition intervention research. The content advisor reviewed and provided feedback on the scoping review question and the search strategy, including criteria and search terms.

As scoping reviews do not aim to produce a critically appraised and synthesized answer to a particular question (systematic reviews have a specific and analytical focus of interest, for example the effectiveness of an interest), critically appraising the included articles is generally not conducted. Scoping reviews have a broader scope or research questions, include a variety of research sources, and include descriptive synthesis approaches. The key differences between a scoping review and systematic review are the absence of critical appraisal of the included studies, the lack of synthesis of findings or quantitative analysis, and conducting a certainty of evidence (CoE) assessment.

### 2.1. Eligibility Criteria

A detailed description of the inclusion and exclusion criteria can be found in Table 1. A priori eligibility criteria were categorized based on the mnemonic population, concept, and context as recommended by the Joanna Briggs Institute. The population of this scoping review was limited to adults (>18 years old) who were diagnosed with MASLD with or without overweight or obesity and with or without established disease (type 2 diabetes, type 1 diabetes, hypertension, cardiovascular disease, hyperlipidemia, or insulin resistance). The concept related to nutrition intervention, a step in the Nutrition Care Process framework, and the context where the intervention was offered were not restricted in the search plan. As it is not necessary to specify outcomes for a scoping review, those were not restricted in the search plan.

### 2.2. Search Strategy

An electronic search of multiple databases like Medline, CINAHL, Cochrane Central Register of Controlled Trials, Cochrane Database of Systematic Reviews, Food Science Source, and SPORTDiscus was conducted by an information specialist. The results were limited by English language and publication year January 2000 to 12 October 2023. The results were deduplicated in EndNote Software version 9.3.3. The search strategy was designed to identify studies on both dietary pattern interventions and bioactive substance interventions. This paper specifically focuses on findings related to bioactive substances. A sample of the search strategy for one database is available in Supplemental Table S1.

### 2.3. Article Selection and Data Extraction

The results from the database search were uploaded onto Rayyan (https://new.rayyan.ai/, accessed on 20 November 2024) [9], a title/abstract screening software. Two-step screening was conducted by two reviewers. All titles/abstracts were individually reviewed by two reviewers, and discrepancies in decisions were settled through discussions and consensus or by a third reviewer. All included articles in this phase were exported onto a Microsoft Excel template designed by the reviewers. After the full-text review by two reviewers, all studies meeting the inclusion criteria as described in the search strategy were marked as include and moved into the data extraction phase. A data extraction template was designed to extract study characteristics and was approved by the content expert. The extracted data included the publication information, location of the study, study population, study purpose, type of intervention (supplement), intervention details, and outcomes.

### 2.4. Synthesis of Results

The study selection process is documented in a PRISMA flowchart. The characteristics and results of the included studies are narratively described, and the results are also represented in charts, figures, and a heat map. As is customary with scoping reviews, critical appraisal of study quality was not conducted. 

## 3. Results

**Figure 1:**
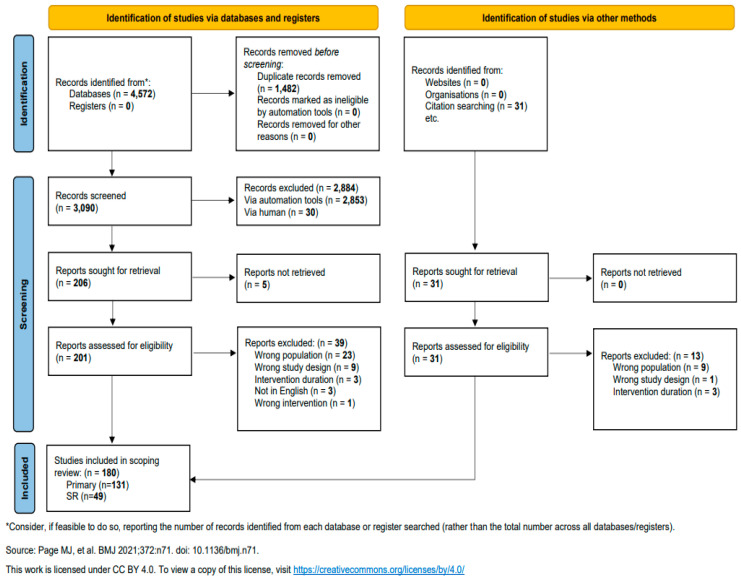
PRISMA flowchart [141] for the bioactive substances in adults with MASLD scoping review.

The initial literature search yielded 4572 records from multiple databases. After removing duplicates, 201 unique records were screened for eligibility (Figure 1). Of these, 131 primary studies and 49 systematic reviews were included in the scoping review. Five of the primary studies were secondary analyses. Eighty-five [10,11,12,13,14,15,16,17,18,19,20,21,22,23,24,25,26,27,28,29,30,31,32,33,34,35,36,37,38,39,40,41,42,43,44,45,46,47,48,49,50,51,52,53,54,55,56,57,58,59,60,61,62,63,64,65,66,67,68,69,70,71,72,73,74,75,76,77,78,79,80,81,82,83,84,85,86,87,88,89,90,91,92,93,94] of the included primary studies reported at least three primary articles for each bioactive substance and forty-six studies [95,96,97,98,99,100,101,102,103,104,105,106,107,108,109,110,111,112,113,114,115,116,117,118,119,120,121,122,123,124,125,126,127,128,129,130,131,132,133,134,135,136,137,138,139,140] reported less than three articles for each bioactive substance. (Table 2) All included studies were randomized controlled trials or controlled trials. In the majority of included trials, the intervention duration was three months (n = 65), followed by two months (n = 31), six months (n = 19), and twelve months or more (n =2). The shortest trials (two each) were 4 and 6 weeks in length, and the remaining nine trials ranged between 10 weeks and 4 months.

**Figure 2:**
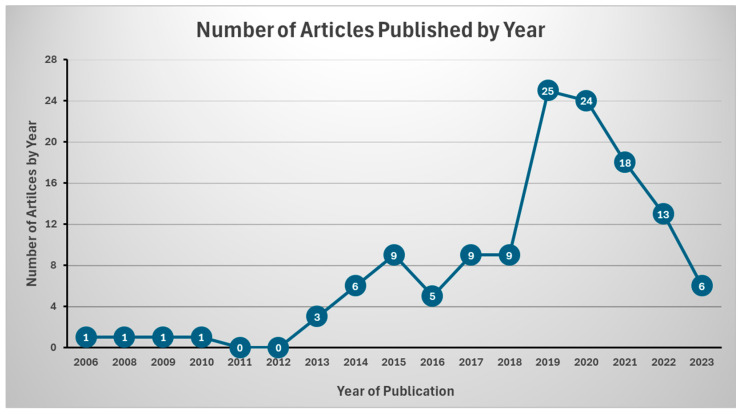
Number of articles meeting inclusion criteria according to publication year.

Our literature search revealed a greater number of publications from the year 2015 onwards. Interest in bioactives and MASLD reached a peak in 2019, with 25 published articles (Figure 2). The majority of the studies were conducted in Iran (n = 92) [12,13,14,15,17,21,22,23,24,25,32,33,34,35,36,38,39,40,43,44,45,46,47,48,49,51,52,53,54,55,56,57,58,59,61,63,64,65,66,67,68,71,72,73,74,75,76,77,79,80,81,82,83,84,85,86,88,92,93,94,95,96,98,99,100,103,107,108,109,110,111,112,113,116,118,119,122,123,125,126,127,128,129,130,131,132,133,135,136,137,138,139], Italy (n = 13) [10,19,20,26,27,28,29,30,60,101,102,106], China (n = 6) [18,31,91,115,134,140], Denmark (n = 2) [37,70], Spain (n = 2) [11,50], the United States (n = 2) [62,69], Korea (n = 2) [114,124], and Taiwan (n = 2) [104,105], and one study each in Australia [16], Cuba [117], Greece [89], Japan [78], Pakistan [42], Malaysia [90], Taiwan [105], and Thailand [120].

### 3.1. Population Characteristics

The majority of the articles included both sexes (n = 124), except one study that focused on males only (n = 1), and six studies that focused on women only. Almost half of the included studies had individuals who were obese (n = 60) [11,13,14,16,18,22,25,31,32,33,34,37,38,39,40,41,44,45,47,50,54,58,59,62,63,68,69,70,71,74,76,78,83,88,90,91,98,99,102,103,104,105,109,110,112,113,114,115,116,118,121,124,128,132,134,135,136,138,140], overweight (n = 31) [12,23,24,26,28,29,30,36,42,46,49,56,57,60,65,66,72,77,82,84,86,89,93,94,97,100,117,122,126,129,130], or extremely obese (n = 4) [15,55,74,75], and five articles [61,67,81,92,111] did not report on sample weight status. Thirty articles included both overweight and obese individuals (n = 30) [10,17,19,20,21,27,35,43,48,51,52,53,64,79,80,85,87,95,96,101,107,108,119,120,125,127,131,133,137,139].

While individuals with MASLD (n = 124) [10,11,12,13,14,15,16,17,18,19,20,21,22,23,24,25,27,28,29,31,32,33,34,35,37,38,39,40,41,42,43,44,45,46,47,48,49,50,51,52,53,54,55,56,57,58,59,60,61,63,64,65,66,67,68,69,70,71,72,73,74,75,76,77,78,79,80,81,82,83,84,85,87,88,89,90,91,92,93,94,95,96,97,98,99,100,101,102,103,105,106,107,108,109,110,111,112,113,114,115,116,117,118,119,120,121,122,123,124,125,126,127,128,129,130,131,132,133,134,135,136,137,138,139,140] and MASH (n = 7) [26,30,36,62,86,104] were the primary focus of the studies, five studies (n = 5) [47,48,60,91,125] also enrolled those with type 2 diabetes (T2D), T2D and impaired glucose tolerance (n = 1) [91], or metabolic syndrome (n = 2) [87,101]. The majority of the articles (n = 113) did not report comorbidities among the included individuals. However, 12 of the studies reported type 2 diabetes [15,20,47,48,51,60,66,85,91,101,102,123], hypertension (n = 7) [28,51,66,101,102,117,123], hyperlipidemia (n = 7) [28,51,66,101,102,117,123], and impaired fasting glucose (n = 2) [91,134] and metabolic syndrome (n = 2) [87,101] as comorbidities in their populations.

### 3.2. Interventions

**Figure 3:**
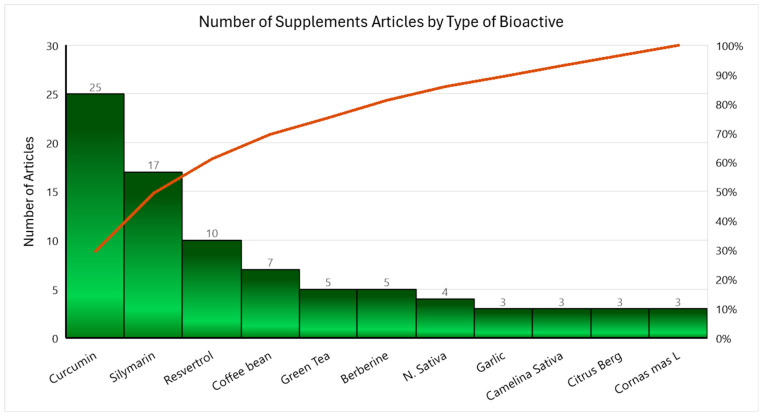
Number of articles meeting inclusion criteria according to type of bioactive substance (bioactive reported in ≥3 articles) (n = 85).

Of 131 articles meeting the eligibility criteria for this scoping review, the most researched bioactive substances were Curcumin (Turmeric) (n = 25) [17,30,32,33,34,35,43,44,51,52,53,54,56,61,63,65,66,67,71,73,74,75,76,77,84], Silymarin (Milk Thistle) (n = 17) [10,11,12,15,19,20,26,27,36,49,55,57,62,69,86,87,90], Resveratrol (n = 10) [13,14,16,18,23,24,25,37,70,89], Coffee (n = 7) [38,39,40,47,48,68,83], Green Tea (n = 5) [41,42,50,78,88], Berberine (n = 5) [31,64,91,93,94], Nigella sativa (n = 4) [21,22,46,72], Camelina sativa (n = 3) [45,58,59], Citrus bergamia (n = 3) [28,29,60], *Cornus mas* L. (n = 3) [81,82,92], and Garlic (n = 3) [79,80,85] (Figure 3). Additionally, there were 46 articles reporting on 36 other bioactive substances with 2 or fewer articles each [93,94,95,96,97,98,99,100,101,103,104,105,106,107,108,109,110,111,112,113,114,115,116,117,118,119,120,121,122,123,124,125,126,127,128,129,130,131,132,133,134,135,136,137,138,139,140]. These included Green Cardamom, Cinnamon, Rosemary Leaf, Cranberry, Licorice Root, Ginger, Masthia, and many others. The primary method of intervention delivery was capsules (n = 92) and tablets (n = 21), followed by powder (n = 5), liquid (n = 4), oil, sachets, or infused in water (n = 3 each). Four articles did not report the delivery method, and some articles used more than one type of delivery method for their bioactive substances.

**Figure 4:**
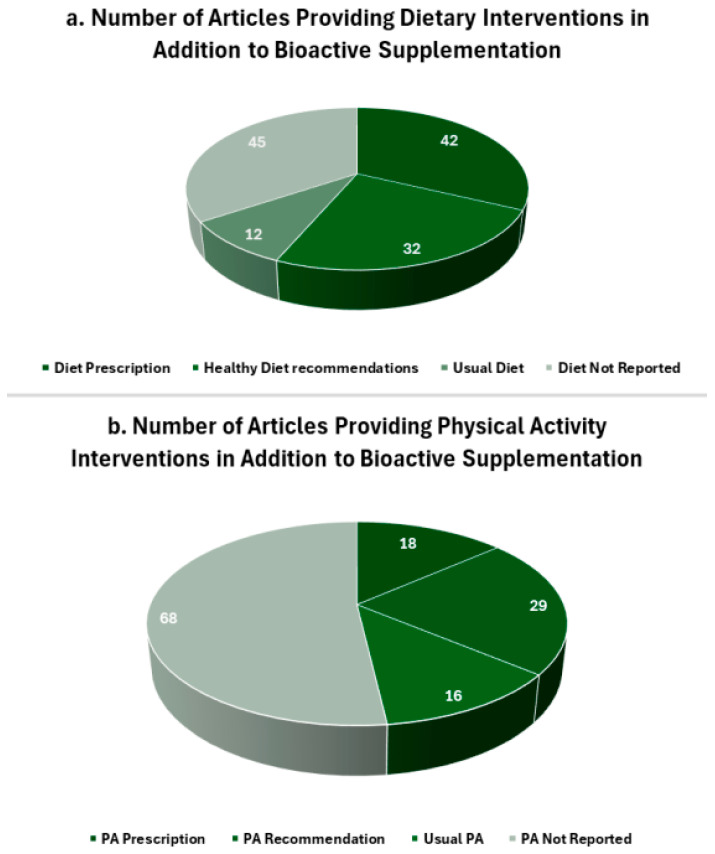
Number of included articles providing dietary (**a**) and physical activity (**b**) co-interventions in addition to bioactive supplementation.

Most of the included articles incorporated additional co-interventions alongside active bioactive supplementation. Diets were prescribed in 42 articles [10,11,12,15,19,23,24,28,29,30,31,35,44,45,49,50,51,58,59,64,73,75,86,87,88,89,91,100,102,105,109,112,113,116,117,122,126,128,131,133,135,136], 32 included recommendations for a healthy diet [14,21,22,39,56,57,61,62,63,65,66,67,77,83,90,97,98,99,100,104,105,107,108,119,121,125,129,130,132,137,138,139], 12 described adherences to a usual diet [16,18,20,27,46,48,60,94,111,114,118,127], and 45 did not provide any details regarding dietary interventions (Figure 4a). The most prescribed diets were calorie restricted (n = 30) [11,12,23,24,35,44,45,49,50,51,58,59,73,75,86,88,91,96,109,112,113,116,117,122,126,128,131,133,135,136], low fat diet (n = 4) [31,64,89,106], Mediterranean (n = 3) [30,87,103], or combination Mediterranean and low calorie (n = 5) [10,19,28,29,101]. Physical activity was prescribed in 18 articles [11,23,24,31,35,44,49,51,56,64,73,75,86,88,91,115,131,133] and encouraged in 29 articles [10,21,22,39,57,61,65,66,67,74,77,83,87,90,96,97,99,100,103,105,107,108,119,122,125,130,132,137,138], adherence to usual physical activity was described in 16 articles [16,20,27,45,46,48,50,58,59,60,103,106,111,116,118,122,127], and 68 articles did not provide any information regarding exercise interventions (Figure 4b).

### 3.3. Systematic Reviews

Forty-nine systematic reviews met the eligibility criteria for this scoping review (Table 3) [142,143,144,145,146,147,148,149,150,151,152,153,154,155,156,157,158,159,160,161,162,163,164,165,166,167,168,169,170,171,172,173,174,175,176,177,178,179,180,181,182,183,184,185,186,187,188,189,190]. Of these, two were umbrella reviews [158,182] and one was a network meta-analysis [176]. Majority of the systematic reviews searched at least two databases (n = 43) [143,144,145,146,147,148,149,150,151,152,153,154,155,156,157,158,160,161,162,163,164,165,166,167,168,169,170,171,172,173,174,175,176,177,178,179,180,181,182,183,184,185,186,187,188,189,190], conducted risk of bias assessments of included articles (n = 43) [142,143,144,145,147,148,149,150,151,152,153,154,155,156,157,158,159,160,161,162,163,164,166,167,168,169,170,172,174,175,176,178,180,181,182,183,184,185,186,187,188,189,190], and followed PRISMA guidelines of publication (n = 39) [142,143,144,145,146,147,148,149,150,151,152,153,154,155,156,157,158,159,160,161,162,165,166,167,168,170,172,174,175,176,178,182,183,185,186,187,188,189,190]. However, only 20 systematic reviews registered their protocols (n = 20) (PROSPERO or any other registration) [147,148,149,152,155,156,157,158,161,166,167,168,170,172,175,176,182,187,188,190] and only 4 systematic reviews evaluated the certainty of evidence for each reported outcome [144,166,182,190]. Of these 49 systematic reviews, 13 systematic reviews focused on Curcumin [144,148,152,153,154,157,161,166,176,178,179,187,188], 12 focused on Coffee or Tea (including catechin extracts) [142,143,145,150,158,162,163,164,171,172,174,180], 10 on bioactive substance combinations [147,149,159,160,170,173,175,181,185,190], 6 on Resveratrol [146,151,169,177,182,183], and 2 others on Silymarin [155,184] and Artichoke Leaf [156,165]. Other bioactive substances with one systematic review each included Ginger [186], Soy [189], Garlic [168], and Caper fruit [167]. Of the 49 included systematic reviews, 40 included randomized control trials or controlled trials [142,143,144,146,148,149,151,152,153,154,155,156,157,159,160,161,162,163,165,166,167,168,169,170,173,175,176,177,178,179,181,182,183,184,185,186,187,188,189,190], 1 included randomized and non-randomized control trials [147], and 8 included observational studies [145,150,158,164,171,172,174,180]. While all systematic reviews included bioactive supplementation (e.g., pill or capsule), several also included interventions that were based on food consumption, such as Coffee or Tea as a beverage [145,150,158,171,172,180], Artichoke Leaf juice [156,165], a diet high in soy [189], and pickled Caper fruit [167]. Many systematic reviews also included studies with co-interventions, such as diet, exercise, pharmacological therapies, or vitamin supplementation.

### 3.4. Outcomes Reported in Primary Studies

**Figure 5:**
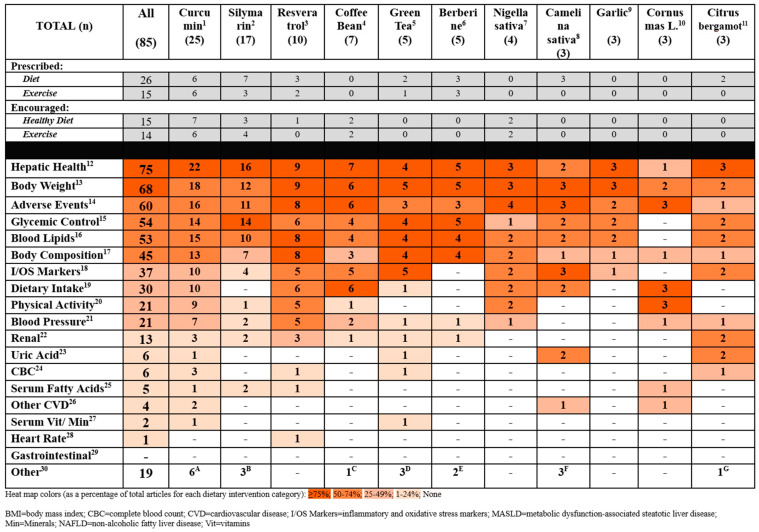
Heat map describing outcomes reported for each bioactive substance with at least n = 3 articles for adults with MASLD. Intervention descriptions: ^1^ Curcumin: Curcumin (n = 9), Nano-curcumin (n = 1), Curcuminoids + Piperine (n = 5), phospholipid Curcumin (n = 1), Phytosomal Curcumin (n = 3), Curcumin Complex + other bioactive substances (nutraceutical) (n = 1), Turmeric (n = 3), Turmeric, Chicory (n = 2); ^2^ Silymarin: Silymarin (n = 9), Silymarin + other substances (n = 8); ^3^ Resveratrol: (n = 10); ^4^ Coffee: Coffee components (n = 2), Green Coffee extract (n = 5); ^5^ Green Tea: Green Tea (n = 1), Green Tea extract (n = 4); ^6^ Berberine: Berberine (n = 2), Berberine + bicyclol (n = 1), Berberis integerrima (n = 1), Berberis aristate + other substances (n = 1); ^7^ *Nigella sativa*: N. Sativa oil (n = 2), N. Sativa (n = 2); ^8^ *Camelina sativa*: (n = 3); ^9^ Garlic: Garlic powder (n = 3); ^10^ *Cornus mas* L: *Cornus mas* L. fruit extract (n = 3); ^11^ *Citrus bergamot*: Bergamot citrus + wild cardoon (n = 1) and Citrus bergamias + Cynara cardunculus (n = 2); Outcome descriptions: ^12^ Hepatic health: includes liver function tests [e.g., alanine aminotransferase (ALT); aspartate aminotransferase (AST); gamma-glutamyl transferase (GGT); alkaline phosphatase (ALP)], various measures of hepatic steatosis and fibrosis [e.g., Controlled Attenuation Parameter (CAP) score, NAFLD grade, etc.], ballooning injury score, liver stiffness, liver volume, and bilirubin. ^13^ Body weight: includes body weight and body mass index. ^14^ Adverse events: includes any self-reported adverse effect of intervention (e.g., nausea, constipation, bloating, etc.) or other adverse event identified by research staff (e.g., biliary disorder, abnormal lab values, etc.). ^15^ Glycemic control: includes blood glucose, total available glucose, hemoglobin A1c, Homeostatic Model Assessment of Insulin Resistance, insulin levels, quantitative insulin sensitivity check index (QUICKI), and C-peptide. ^16^ Blood lipids: includes total cholesterol, high-density lipoproteins, low-density lipoproteins, non-HDL-C, triglycerides, TC:HDL ratio, lipoprotein remnants. ^17^ Body composition: includes body fat, lean body mass, waist, hip and abdominal circumference, waist-to-hip ratio, fat mass, fat-free mass, and visceral fat. ^18^ I/OS markers: inflammatory and oxidative stress markers includes immunological, inflammatory, and oxidative stress markers such as C-reactive protein, interleukin-6, Tumor Necrosis Factor, adipokines and cytokines (leptin, adiponectin, etc.), malondialdehyde (MDA), total antioxidant capacity (TAC), fibroblast growth factor (FGF-21), glutathione peroxidase (GSH), superoxide dismutase (SOD), total glutathione, and many others. ^19^ Dietary intake: includes intake of energy, macro- and micronutrients, antioxidants, fiber, flavonoids, and caffeine. ^20^ Physical activity: includes any physical activity (aerobic, muscle-strengthening, stretching, balance, etc.). ^21^ Blood pressure: includes systolic and diastolic blood pressure. ^22^ Renal: includes renal function lab values (creatinine, blood urea nitrogen, etc.). ^23^ Uric acid: includes uric acid blood test. ^24^ CBC: includes albumin, hematocrit, hemoglobin, neutrophils, white blood cells, platelets, etc. ^25^ Serum fatty acids: includes lipid accumulation product (LAP), serum fatty acid profile (includes phospholipids, saturated and polyunsaturated fatty acids, etc.), and free fatty acids. ^26^ Other CVD: cardiovascular outcomes (other than lipids), including atherogenic index of plasma (AIP), homocysteine, coronary artery calcium (CAC), Castelli risk index I (CRI-I), CRI-II, and atherogenic coefficient (AC). ^27^ Serum vit/min: includes serum vitamin and minerals: 25-hydroxy vitamin D [25(OH)D3], potassium, iron, ferritin, transferrin, and magnesium. ^28^ Heart rate: includes heart rate and pulse. ^29^ Gastrointestinal: includes intestinal permeability and gut microbiota. ^30^ Other: other outcomes: ^A^ frequency of comorbidities, nesfatin, 8-Hydroxy-2′-Deoxyguanosine, methylation in MutL homolog 1, MutS homolog 2 (MSH2), carboxymethyl lisine (CML), 8-hydroxy-2′-deoxyguanosine, urea, liver-to-spleen CT attenuation ratio. ^B^ Thiobarbituric acid reactive substances (n = 2), tissue inhibitor of metalloproteinase-I/II, amino terminal propeptide of type III procollagen, endocan, high mobility group box-1, and thiobarbituric acid reactive substances. ^C^ Thyroid-stimulating hormone. ^D^ Liver-to-spleen computed attenuation ratio, nonprotein RQ, substrate oxidation. ^E^ Liver-to-spleen computed attenuation ratio, urea. ^F^ Mental health: general health questionnaire, depression, anxiety and stress scale. ^G^ Endothelial dysfunction parameters.

Some of the most commonly reported outcomes in the 85 included studies (bioactive substance with at least n = 3 studies) were as follows: hepatic health (e.g., liver function blood tests, measures of hepatic steatosis and fibrosis, liver volume, etc.) [10,11,13,14,15,16,17,18,19,20,21,22,23,24,25,26,27,28,29,30,31,32,33,35,36,37,38,39,40,41,42,43,44,46,47,48,49,51,52,54,55,56,57,58,59,60,61,62,63,64,66,67,68,69,71,73,74,75,76,77,78,79,80,82,83,84,85,86,87,88,89,90,91], body weight [10,11,12,13,14,15,16,17,18,19,20,21,22,23,24,25,26,27,28,29,30,31,33,34,35,36,37,38,39,40,41,42,44,45,46,47,50,51,52,54,56,57,58,59,61,63,64,66,68,70,71,73,74,76,77,78,79,80,81,83,84,85,87,88,90,91,92], adverse events (nausea, bloating, and abnormal lab values) [10,11,12,13,14,15,16,18,21,22,23,24,25,29,30,31,35,36,37,38,39,40,41,42,43,44,45,46,47,48,50,51,52,53,54,55,57,58,59,61,62,64,66,69,71,72,74,75,76,77,80,81,82,83,84,85,87,90,91,92], glycemic control [10,11,12,13,15,16,17,18,19,20,21,24,26,27,28,29,30,31,34,36,37,40,41,42,43,44,45,47,48,49,50,52,59,62,63,64,65,67,69,71,73,76,77,80,82,83,84,85,87,88,89,90,91], blood lipids [10,11,12,13,15,16,17,18,19,21,24,25,28,29,30,31,33,36,37,38,40,41,42,43,44,46,47,48,49,50,51,52,58,59,61,65,67,69,70,71,73,76,77,79,83,84,85,87,88,89,90,91], body composition [10,11,12,13,16,17,18,19,20,21,22,23,24,25,27,29,30,31,33,35,37,41,44,47,50,54,58,60,61,64,66,70,73,74,76,77,78,79,83,84,87,88,91,92], inflammatory markers and oxidative stress markers (e.g., C-reactive protein (CRP), adipokines, total antioxidant capacity (TAC), etc.) [13,14,16,18,20,22,26,27,29,30,32,37,38,39,40,41,42,43,44,45,48,50,52,53,58,59,60,63,69,72,75,76,78,80,82,83,84,86,88], dietary intake [13,14,16,21,22,23,24,25,32,33,35,38,39,44,45,47,48,50,51,54,59,63,68,73,74,77,81,82,83,84,92], physical activity [10,13,14,21,22,23,24,25,32,33,35,56,61,73,77,81,82,83,84,92], and blood pressure. [10,16,18,20,24,25,29,37,39,41,44,48,51,66,72,76,77,84,92,93]. The heat map (Figure 5) illustrates the distribution of outcomes assessed by each bioactive substance (intervention).

Hepatic health outcomes were reported in almost 90% (n = 117) of articles. The most frequently reported were liver function tests [e.g., alanine aminotransferase (ALT); aspartate aminotransferase (AST); gamma-glutamyl transferase (GGT); and alkaline phosphatase (ALP) in 84.5% of articles (n = 111)]. Scoring or grading measures of hepatic steatosis and fibrosis [e.g., Controlled Attenuation Parameter (CAP) score, ballooning injury score NAFLD grade, etc.] and other liver function parameters (e.g., liver stiffness, liver volume, and bilirubin) were reported in 63.4% (n = 83) of articles.

Almost 80% of articles (n = 66) reported body mass index or body weight outcomes. Various types of body composition measures (body fat percentage, lean mass, visceral fat, and waist and hip circumference) were reported in 55% (n = 72) of articles. The most frequently reported body composition outcomes were waist and hip circumference and waist to hip ratio, reported in 50% (n = 65) of articles. Almost three-quarters (n = 95) reported adverse event or side effect outcomes. These included self-reported side effects by subjects (e.g., nausea, constipation, bloating, etc.) or other adverse effects identified by research staff (e.g., biliary disorder, abnormal lab values, etc.).

In terms of cardiometabolic risk indicators, over two-thirds (n = 88) reported glycemic control outcomes, with 62.6% (n = 82) reporting blood glucose or HbA1c, while 48.8% (n = 64) reported on one or more insulin sensitivity markers [Homeostatic Model Assessment of Insulin Resistance (HOMA-IR), insulin levels, and quantitative insulin sensitivity check index (QUICKI)]. One or more blood lipids (total cholesterol, high- or low-density lipoproteins, triglycerides, etc.) were reported in 66.4% (n = 87) of articles. Only about one-fourth of articles (n = 30) reported blood pressure outcomes.

Almost half (n = 60) of the articles reported inflammatory and oxidative stress markers such as CRP, interleukin-6 (IL-6), Tumor Necrosis Factor (TNF), adipokines and cytokines (leptin, adiponectin, etc.), malondialdehyde (MDA), TAC, fibroblast growth factor (FGF-21), glutathione peroxidase (GSH), superoxide dismutase (SOD), total glutathione, and many others.

About one-third of articles reported energy or macronutrient intake. Caloric intake was reported in 35% (n = 47) of articles, followed by carbohydrate, fat, and protein intake at 32% (n = 42).

### 3.5. Funding

The majority of the included articles were funded by universities (n = 74) [12,13,14,15,17,21,22,25,33,34,35,37,38,39,40,43,44,45,46,52,53,54,55,57,59,63,65,66,68,71,73,74,75,76,77,81,82,83,85,86,89,90,92,93,94,96,98,99,100,104,107,108,110,111,112,113,116,118,119,120,122,123,126,127,128,129,130,131,132,133,134,137,139,140], governments (n = 23) [16,18,24,28,29,37,47,48,50,62,70,78,79,80,84,91,98,103,115,120,121,124,140], industry (n = 9) [27,30,37,62,65,66,69,94,110], foundations (n = 8) [16,23,24,37,70,71,115,140], hospitals (n = 5) [67,70,105,106,134], or were not funded (n = 5) [19,31,42,56,102], and nineteen studies did not report the funding source.

## 4. Discussion

The objective of this scoping review was to identify and characterize the current evidence on dietary bioactive-substance-based interventions for adults, 18 years of age or older, with MASLD/NAFLD published from January 2000 to 12 October 2023. This scoping review identified 131 primary studies, which included RCTs and controlled trials, along with 49 SRs, underscoring the growing body of research on MASLD/NAFLD dietary bioactive interventions, particularly since 2015. Most studies focused on dietary bioactive substances, such as Curcumin, Silymarin, and Resveratrol, highlighting these dietary bioactive substances’ potential roles in managing MASLD/NAFLD, which include suggested mechanisms of action such as antioxidant activity, anti-inflammatory and antifibrotic effects, the regulation of lipid metabolism, and modulation of the gut–liver axis [191]. These findings demonstrate an increased interest in dietary bioactive substances for improving hepatic health and addressing associated metabolic dysfunctions [3]. However, the heterogeneity in study populations, interventions, and reported outcomes presents challenges to drawing definitive conclusions.

One of the significant observations from this scoping review is the geographical concentration of research, with the majority of studies conducted in Iran. While this focus provides valuable insights, it raises questions about the generalizability of findings to diverse populations. Liver diseases, MASLD, hepatitis, and cirrhosis are major public health challenges in Iran. The increasing prevalence of MASLD is largely driven by rising rates of obesity, metabolic syndrome, and type 2 diabetes. Additionally, hepatitis B and C remain endemic in certain regions, contributing to the burden of liver disease [192,193]. Limited representation from others, such as Asia, North America, Latin America, and Europe, suggests a need for more geographically diverse research to ensure broader applicability.

Another finding is the frequent inclusion of co-interventions, such as dietary prescriptions or recommendations for a healthy diet, in many primary studies. While this reflects the multifaceted nature of MASLD/NAFLD management, it complicates the ability to isolate the effects of dietary bioactive supplementation. Isolating the effects of bioactive substances from concomitant lifestyle interventions is a significant challenge in research. This complexity arises from the multifaceted nature of human behavior, physiology, and the interactions between interventions. Confounding variables present a major hurdle, as overlapping effects from lifestyle changes, such as physical activity or dietary interventions, may mimic or enhance the outcomes of bioactive substances. Behavioral changes, like healthier habits adopted alongside supplementation, further obscure the attribution of observed effects. Blinding, variability in individual responses, and measurement challenges may occur. To address these challenges, strategies such as study designs using factorial or crossover approaches, objective measurements through wearable devices and biomarkers, and advanced statistical methods for controlling confounders and interactions may be applied. Monitoring adherence to interventions and conducting long-term studies can also help distinguish and disentangle the independent and combined effects of bioactive substances and lifestyle between immediate and delayed effects. By addressing these complexities, research can better interventions [194,195]. Future research should aim to better determine the independent and synergistic effects of these interventions to inform clinical recommendations and practice.

Interestingly, nearly half of the included primary studies did not report on subjects’ comorbidities, despite MASLD/NAFLD often occurring in the context of metabolic disorders such as type 2 diabetes and hypertension. This lack of detail limits the ability to understand the impact of interventions in more complex clinical scenarios, highlighting an important gap in the literature. Research that explicitly targets populations with comorbidities or stratifies outcomes based on these conditions is necessary to develop tailored dietary bioactive intervention strategies.

Among the wide range of outcomes reported in the primary studies, the most researched included hepatic health (liver function tests, imaging tests, biopsies, etc.), body weight and body composition, inflammatory and oxidative stress markers, and biomarkers of cardiometabolic risk, such as glycemic control and blood lipids. Energy and macronutrient intake was also frequently reported. Regarding safety data for dietary bioactive substances, adverse events were evaluated in 72% of the primary studies. Few primary studies reported on incident events, CVD development, or mortality due to the challenges of conducting long-term research with RCTs.

The SRs included in this scoping review provided additional insights but revealed methodological inconsistencies. While most SRs adhered to PRISMA guidelines and conducted risk of bias assessments, fewer registered their protocols or evaluated the certainty of evidence. This inconsistency underscores the need for higher methodological standards in future SRs to strengthen the reliability of evidence in dietary bioactive substance research. Yates et al. established a comprehensive framework for determining recommended intakes of dietary bioactive substances that provide health benefits. This framework should be used to guide recommendations as it is based on utilizing high-quality scientific evidence that thoroughly evaluates efficacy and safety by qualified experts. Moreover, the framework supports only making quantified recommendations with at least moderate-quality evidence [2].

The scoping review’s strength lies in its use of a rigorous scientific approach to explore the impact of dietary bioactive substances in adults with MASLD/NAFLD. The protocol for this project adhered to the PRISMA checklist for scoping reviews and is registered on Open Science Framework. Additional strengths include the involvement of a content expert, who was consulted throughout all stages of the review to ensure accurate and relevant data and reporting, as well as the contribution of an information specialist who conducted a broad and in-depth literature search across six databases to comprehensively identify studies on dietary bioactive substances related to MASLD/NAFLD.

However, some limitations should be acknowledged. The restriction to English-language articles may have excluded relevant studies published in other languages. Additionally, the absence of critical appraisal of the included studies’ methodological quality as well as a lack of synthesis of findings or quantitative analysis and assessment of certainty of evidence (CoE), as is customary for scoping reviews, limits the ability to assess the robustness of individual findings [4,5,196]. Furthermore, the broad inclusion criteria and diverse interventions captured in the review may introduce variability that limits the synthesis of specific conclusions.

The findings from this scoping review highlight several opportunities for future research. First, there is a need for more standardized reporting of study designs, subject characteristics, and outcomes. Consistency in defining and reporting MASLD and its comorbidities will enhance the comparability of findings across studies. Second, while this review identified several articles related to dietary bioactive substance intake, further high-quality RCTs with rigorous methodologies are needed to establish their efficacy and safety. Third, the integration of interventions with lifestyle modifications, such as diet and physical activity, requires more focused investigation to determine the optimal combination for managing MASLD. Finally, as MASLD research evolves, the incorporation of outcomes related to quality of life, healthcare utilization, and cost effectiveness will be critical for translating findings into clinical practice and policy.

## 5. Conclusions

In conclusion, this scoping review provides a detailed mapping of the current evidence on dietary bioactive-substance-based interventions for MASLD/NAFLD, highlighting potential areas of research and critical gaps. By integrating findings from primary studies and SRs, this review underscores the potential of nutrition-based approaches in MASLD management while identifying areas for improvement in study design and reporting. These insights will help guide future research and inform evidence-based strategies to address the growing burden of MASLD.

## Figures and Tables

**Table 1:** Eligibility criteria for scoping review examining availability of literature describing bioactive substance interventions among adults with MASLD (previously NAFLD).

PICO	Inclusion Criteria	Exclusion Criteria
Population	Free-living adults (>18 years old) With metabolic dysfunction-associated steatotic liver disease (MASLD, previously NAFLD) MASLD/NAFLD (absence of significant alcohol use and other causes of hepatic steatosis) with or without overweight or obesity and with or without established disease (T2DM, T1DM, HTN, CVD, hyperlipidemia, or insulin resistance) NASH	Nonadults < 18 years (adolescents, children, and infants) Institutionalized individuals (i.e., inmates and patients of mental disease institutions) Pregnant, postpartum, or lactating women Established or diagnosed alcoholic fatty liver disease Significant alcohol use and other causes of hepatic steatosis (SLD)
Intervention	Any interventions (e.g., behavioral counseling, nutrition education, dietary prescription, etc.) that are provided by a healthcare provider (dietitian, nutritionist, physician, or nurses) Carbohydrate- and fructose-restricted diet Hypocaloric diets Fat-restricted diets Med diet DASH diet Intermittent fasting Dietary patterns (vegetarian/vegan) High polyphenol diets (Coffee and Tea) Bioactives MNT Micronutrients (Vitamin E)	Interventions that included fasting (enteral feedings or parenteral nutrition) Intervention length < 4 weeks
Comparison	At least one comparator group (e.g., usual diet or another contrasting diet)	No control group or comparator diet
Outcomes	Any	Does not report any outcomes of interest
Limits		
Study Designs	RCTs and controlled clinical trials Cohort studies Systematic reviews; meta-analyses Guidelines Relevant systematic reviews will be searched for potentially included articles by the database search	Case–control, cross-sectional, before–after studies, ecological studies, single case study, case report, case series, or non-comparative Letters to the editor/commentary, poster session, abstract, or study protocol
Age range	Adults > 18 years old	
Date range	January 2000 to 12 October 2023	
Language	English only	
Databases	MEDLINE, CINAHL, Cochrane Database of Systematic Reviews, Cochrane CENTRAL, Food Science Source, and SportDiscus	

**Table 2:** Bioactive substances reported in articles meeting the inclusion criteria.

At Least 3 Articles per Bioactive (n = 85)	Less Than 3 Articles per Bioactive (n = 46)
Berberine (berberis) Camelina sativa Citrus bergamia Coffee Bean *Cornus mas* L. Fruit Curcumin (Turmeric) Nigella sativa Resveratrol Silymarin (Milk Thistle) Garlic Green Tea	Anthocyanin (from bilberry and black current) Antrodia cinnamomea Mycelium (mushroom) Artemisia anuua I. Artichoke Leaf Beta cryptoxanthin Beta vulgaris (beet) Chlorella vulgaris (algae) Cinnamon Cranberry D-002 (Beeswax Alcohol) Fenugreek Flaxseed Fructus akebiae Fucoidan, Fucoxanthin Genistein Ginger Grape Seed Green Cardamom Gynostemma pentaphyllum Hesperidin Hydroxycitric acid Licorice Root Mastiha Naringenin Nutraceutical mixture Oligonol (Litchi-derived polyphenol) Pinitol (polyol) Plantago major seed Pomegranate Propolis Purslane Rosemary Saffron Shirazi Thyme Sour Tea Sumac

**Table 3:** Characteristics of relevant systematic reviews or meta-analyses that met the inclusion criteria.

	ALL	Curcumin	Silymarin	Resveratrol	Coffee/Tea Extracts	Multiple Bioactives ^A^	Garlic	Ginger	Artichoke Leaf	Caper Fruit	Soy/Genestein
N	49	13	2	6	12	10	1	1	2	1	1
Study Designs Included in SRs											
RCT	40	13 ^B^	2	6 ^C^	4	9	1	1	2	1	1 ^C^
RCT, NRCT	1					1					
OBS	8				8 ^D,E^						
Quality of SR											
≥2 databases	47	13	2	6	11	9	1	1	2	1	1
PROSPERO	20	8	1	1	2	5	1	0	1	1	0
PRISMA	39	11	1	5	8	8	1	1	2	1	1
ROB	43	11	2	5	11	9	1	1	1	1	1
QOE/GRADE	4	2	0	1	0	1	0	0	0	0	0

GRADE = Grading of Recommendations, Assessment, Development, and Evaluations; NRCT = non-randomized controlled trial(s); OBS = observational studies; PRISMA = Preferred Reporting Items for Systematic Reviews and Meta-Analyses; QOE = quality of evidence; RCT(s) = randomized controlled trial(s); ROB = risk of bias; SR = systematic review(s). ^A^ More than one individual bioactive substance or combinations of two or more bioactive ingredient supplements (e.g., proprietary blends). ^B^ Includes one network meta-analysis of RCTs. ^C^ Includes one umbrella review of SRs with RCTs. ^D^ Includes one umbrella review of SRs with observational studies. ^E^ Includes two SRs that did not specify eligible study designs but included only SRs of observational studies in results.

## Data Availability

No new data were created or analyzed in this study as all extracted data was available in included articles. Data sharing is not applicable to this article.

## Supplementary Materials

The following supporting information can be downloaded at https://www.mdpi.com/article/10.3390/nu17030453/s1, Table S1: Search Strategy Sample for MASLD Scoping Review from MEDLINE (via EbSCO), File S1: Preferred Reporting Items for Systematic Reviews and Meta-Analyses extension for Scoping Reviews (PRISMA-ScR) checklist.
