# Supplementary material for: Evaluating Bioactive-Substance-Based Interventions for Adults with MASLD: Results from a Systematic Scoping Review
Source: Nutrients. 2025 Jan 26;17(3):453. doi: 10.3390/nu17030453 (PMC11820841; doi:10.3390/nu17030453)
Supplement: Supplementary file 1 [file nutrients-17-00453-s001.zip › nutrients-3404214-supplementary Table S1.pdf]

Supplementary File Table S1: Search Strategy Sample for MASLD Scoping Review from MEDLINE (via EbSCO)

MEDLINE (Ebsco)

#	Query	Limiters/Expanders	Last Run Via	Results
S31	S30 NOT TI (mice OR mouse OR murine* OR rabbit* OR lapine* OR rat OR rats OR rodent* OR pig OR pigs OR piglet* OR swine OR porcine* OR canine* OR dog OR dogs OR horse* OR equine OR cow OR cattle OR bovine* OR sheep OR ovine* OR broiler* OR veterinar* OR hens OR "in vitro" OR "in vivo" OR "animal model" or "animal models")	Search modes - Boolean/Phrase	Interface - EBSCOhost Research Databases Search Screen - Advanced Search Database - MEDLINE Complete	2,094
S30	S29 AND DT 20000101-20241231	Search modes - Boolean/Phrase	Interface - EBSCOhost Research Databases Search Screen - Advanced Search Database - MEDLINE Complete	2,959
S29	S28 AND LA English	Search modes - Boolean/Phrase	Interface - EBSCOhost Research Databases Search Screen - Advanced Search Database - MEDLINE Complete	2,965
S28	S27 NOT PT (comment or editorial or news or newspaper article)	Search modes - Boolean/Phrase	Interface - EBSCOhost Research Databases Search	3,135

			Screen - Advanced Search Database - MEDLINE Complete	
S27	S26 NOT MH (("child+" OR "infant+" or "adolescent") NOT ("adult+"))	Search modes - Boolean/Phrase	Interface - EBSCOhost Research Databases Search Screen - Advanced Search Database - MEDLINE Complete	3,162
S26	S19 AND S25	Search modes - Boolean/Phrase	Interface - EBSCOhost Research Databases Search Screen - Advanced Search Database - MEDLINE Complete	3,309
S25	S20 OR S21 OR S22 OR S23 OR S24	Search modes - Boolean/Phrase	Interface - EBSCOhost Research Databases Search Screen - Advanced Search Database - MEDLINE Complete	6,455,568
S24	PT (guideline or "practice guideline" or "consensus development conference" or "consensus development conference, NIH") OR TI (guideline* or standards or	Search modes - Boolean/Phrase	Interface - EBSCOhost Research Databases Search	325,780

	consensus* or recommendat*) OR TI ("practice parameter*" or "position statement*" or "policy statement*" or CPG or CPGs or "best practice*") OR TI (care N1 (path or paths or pathway or pathways or map or maps or plan or plans or standard)) OR TI ((critical or clinical or practice) N1 (path or paths or pathway or pathways or protocol*)) OR TI (algorithm* and (pharmacotherap* or chemotherap* or chemotreatment* or therap* or treatment* or intervention*)) OR TI (algorithm* and (screening or examination or test or tested or testing or assessment* or diagnosis or diagnoses or diagnosed or diagnosing)) OR AU (guideline* or standards or consensus* or recommendat*) OR ((TI ("systematic review") OR PT ("systematic review") OR CI ("systematic review") OR MH ("systematic review")) AND (TI ("practice guideline*" or "treatment guideline*" or "clinical guideline*" or "guideline recommendation*") OR AB ("practice guideline*" or "treatment guideline*" or "clinical guideline*" or "guideline recommendation*") OR CI ("practice guideline*" or "treatment guideline*" or "clinical guideline*" or "guideline recommendation*")))		Screen - Advanced Search Database - MEDLINE Complete	
S23	PT ("systematic review" OR "meta analysis") or MH ("systematic reviews as topic+" OR "meta-analysis as topic+" OR " Technology assessment, biomedical+") or TI (meta-analy* or metanaly* or metaanaly* or met analy* or integrative research or integrative review* or integrative overview* or research integration or research overview* or collaborative review* or systematic review* or	Search modes - Boolean/Phrase	Interface - EBSCOhost Research Databases Search Screen - Advanced Search Database - MEDLINE Complete	595,316

	systematic overview* or evidence-based review* or evidence-based overview* or (evidence N2 (review* or overview*)) or meta-review* or meta-overview* or meta-synthes* or rapid review* or "review of reviews" or umbrella review? or technology assessment* or HTA or HTAs or (network N0 (meta-analy* or metanaly* or metaanaly* or met analy*)) or (network N0 (MA or MAs)) or NMA or NMAs or MTC or MTCs or MAIC or MAICs or "indirect* compar*" or (indirect treatment* N0 compar*) or (mixed treatment* N0 compar*) or (multiple treatment* N0 compar*) or (multi-treatment* N0 compar*) or "simultaneous* compar*" or "mixed comparison#") or AB (meta-analy* or metanaly* or metaanaly* or met analy* or integrative research or integrative review* or integrative overview* or research integration or research overview* or collaborative review* or systematic review* or systematic overview* or evidence-based review* or evidence-based overview* or (evidence N2 (review* or overview*)) or meta-review* or meta-overview* or meta-synthes* or rapid review* or "review of reviews" or umbrella review? or technology assessment* or HTA or HTAs or (network N0 (meta-analy* or metanaly* or metaanaly* or met analy*)) or (network N0 (MA or MAs)) or NMA or NMAs or MTC or MTCs or MAIC or MAICs or "indirect* compar*" or (indirect treatment* N0 compar*) or (mixed treatment* N0 compar*) or (multiple treatment* N0 compar*) or (multi-treatment* N0 compar*) or "simultaneous* compar*" or "mixed			

	comparison#") or CI (meta-analy* or metanaly* or metaanaly* or met analy* or integrative research or integrative review* or integrative overview* or research integration or research overview* or collaborative review* or systematic review* or systematic overview* or evidence-based review* or evidence-based overview* or (evidence N2 (review* or overview*)) or meta-review* or meta-overview* or meta-synthes* or rapid review* or "review of reviews" or umbrella review? or technology assessment* or HTA or HTAs or (network N0 (meta-analy* or metanaly* or metaanaly* or met analy*)) or (network N0 (MA or MAs)) or NMA or NMAs or MTC or MTCs or MAIC or MAICs or "indirect* compar*" or (indirect treatment* N0 compar*) or (mixed treatment* N0 compar*) or (multiple treatment* N0 compar*) or (multi-treatment* N0 compar*) or "simultaneous* compar*" or "mixed comparison#") or SO (cochrane or health technology assessment or evidence report or systematic reviews)			
S22	PT (controlled clinical trial) or MH ("clinical trial+" or "controlled clinical trials as topic" or "non-randomized controlled trials as topic" or "controlled before-after studies" or "interrupted time series analysis" or "historically controlled study" or "control groups") or TI ((control* N1 trial*) or (nonrandom* or "non-random*" or "quasi-random*" or "quasi-experiment*") or (nRCT or nRCTs or "non-RCT#") or (control* N2 ("before and after" or "before after")) or "time series" or (pre- N2 post-) or (pretest N2 posttest) or (control* N1	Search modes - Boolean/Phrase	Interface - EBSCOhost Research Databases Search Screen - Advanced Search Database - MEDLINE Complete	1,908,624

	stud*) or (control* N1 group*)) or AB ((control* N1 trial*) or (nonrandom* or "non-random*" or "quasi-random*" or "quasi-experiment*") or (nRCT or nRCTs or "non-RCT#") or (control* N2 ("before and after" or "before after")) or "time series" or (pre- N2 post-) or (pretest N2 posttest) or (control* N1 stud*) or (control* N1 group*)) or CI ((control* N1 trial*) or (nonrandom* or "non-random*" or "quasi-random*" or "quasi-experiment*") or (nRCT or nRCTs or "non-RCT#") or (control* N2 ("before and after" or "before after")) or "time series" or (pre- N2 post-) or (pretest N2 posttest) or (control* N1 stud*) or (control* N1 group*)) or TI (trial)			
S21	MH ("Cohort Studies" OR "Longitudinal Studies" OR "Prospective Studies") OR PT ("Comparative Study") OR TI (cohort* OR longitudinal* OR prospective*) OR AB (cohort* OR longitudinal* OR prospective*) OR CI (cohort* OR longitudinal* OR prospective*) OR TI ((comparative OR comparison) N0 (study or studies)) OR AB ((comparative OR comparison) N0 (study or studies)) OR CI ((comparative OR comparison) N0 (study or studies))	Search modes - Boolean/Phrase	Interface - EBSCOhost Research Databases Search Screen - Advanced Search Database - MEDLINE Complete	3,890,864
S20	PT ("controlled clinical trial" or "randomized controlled trial" or "equivalence trial" or "pragmatic clinical trial") or MH ("clinical trials as topic" or "randomized controlled trials as topic") or TI (randomi#ed or randomi#ation# or randomly or RCT or placebo*) or AB (randomi#ed or randomi#ation# or randomly or RCT or placebo*) or CI (randomi#ed or randomi#ation# or randomly or RCT	Search modes - Boolean/Phrase	Interface - EBSCOhost Research Databases Search Screen - Advanced Search Database - MEDLINE Complete	1,745,819

	or placebo*) or TI ((singl* or doubl* or trebl* or tripl*) N0 (mask* or blind* or dumm*)) or AB ((singl* or doubl* or trebl* or tripl*) N0 (mask* or blind* or dumm*)) or CI ((singl* or doubl* or trebl* or tripl*) N0 (mask* or blind* or dumm*)) or TI (trial)			
S19	S3 AND S18	Search modes - Boolean/Phrase	Interface - EBSCOhost Research Databases Search Screen - Advanced Search Database - MEDLINE Complete	12,147
S18	S4 OR S5 OR S6 OR S7 OR S8 OR S9 OR S10 OR S11 OR S12 OR S13 OR S14 OR S15 OR S16 OR S17	Search modes - Boolean/Phrase	Interface - EBSCOhost Research Databases Search Screen - Advanced Search Database - MEDLINE Complete	1,884,103
S17	TI ("intermittent fasting" OR "alternate day fasting" OR "circadian diet*" OR "circadian fast*") OR AB ("intermittent fasting" OR "alternate day fasting" OR "circadian diet*" OR "circadian fast*") OR CI ("intermittent fasting" OR "alternate day fasting" OR "circadian diet*" OR "circadian fast*")	Search modes - Boolean/Phrase	Interface - EBSCOhost Research Databases Search Screen - Advanced Search Database - MEDLINE Complete	1,626
S16	TI ("polyphenol*" OR "curcumin" OR "resveratrol" OR "naringenin" OR "anthocyanin" OR "hesperidin" OR "catechin" OR "silymarin" OR	Search modes - Boolean/Phrase	Interface - EBSCOhost Research Databases	171,235

	"genistein" OR "coffee" OR "tea") OR AB ("polyphenol*" OR "curcumin" OR "resveratrol" OR "naringenin" OR "anthocyanin" OR "hesperidin" OR "catechin" OR "silymarin" OR "genistein" OR "coffee" OR "tea") OR CI ("polyphenol*" OR "curcumin" OR "resveratrol" OR "naringenin" OR "anthocyanin" OR "hesperidin" OR "catechin" OR "silymarin" OR "genistein" OR "coffee" OR "tea")		Search Screen - Advanced Search Database - MEDLINE Complete	
S15	TI (("calcium" OR "iron" OR "ferritin*" OR "zinc" OR "copper" OR "vitamin*" OR "folate" OR "folic acid" OR "niacin" OR "riboflavin" OR "magnesium" OR "multivitamin*" OR "multi-vitamin*" OR "micronutrient*" OR "micro-nutrient*" OR "multimineral*" OR "multi-mineral" OR "antioxidant*" OR "anti-oxidant*" OR "pre-biotic*" OR "prebiotic*" OR "pro-biotic*" OR "probiotic*") N2 ("diet*" OR "supplement*" OR "food" OR "intake" OR "consumption")) OR AB (("calcium" OR "iron" OR "ferritin*" OR "zinc" OR "copper" OR "vitamin*" OR "folate" OR "folic acid" OR "niacin" OR "riboflavin" OR "magnesium" OR "multivitamin*" OR "multi-vitamin*" OR "micronutrient*" OR "micro-nutrient*" OR "multimineral*" OR "multi-mineral" OR "antioxidant*" OR "anti-oxidant*" OR "pre-biotic*" OR "prebiotic*" OR "pro-biotic*" OR "probiotic*") N2 ("diet*" OR "supplement*" OR "food" OR "intake" OR "consumption")) OR CI (("calcium" OR "iron" OR "ferritin*" OR "zinc" OR "copper" OR "vitamin*" OR "folate" OR "folic acid" OR "niacin" OR "riboflavin" OR "magnesium" OR "multivitamin*" OR "multi-vitamin*" OR "micronutrient*" OR "micro-nutrient*" OR	Search modes - Boolean/Phrase	Interface - EBSCOhost Research Databases Search Screen - Advanced Search Database - MEDLINE Complete	123,544

	"multimineral*" OR "multi-mineral" OR "antioxidant*" OR "anti-oxidant*" OR "pre-biotic*" OR "prebiotic*" OR "pro-biotic*" OR "probiotic*") N2 ("diet*" OR "supplement*" OR "food" OR "intake" OR "consumption"))			
S14	TI (("fat" OR "carb*" OR "calorie*" OR "gluten" OR "wheat" OR "fiber" OR "fibre" OR "protein" OR "sodium" OR "salt" OR "fructose" OR "polyphenol") N3 ("free" OR "restrict*" OR "low" OR "high" OR "without" OR "eliminate*") N3 ("diet*" OR "food*" OR "eat*")) OR AB (("fat" OR "carb*" OR "calorie*" OR "gluten" OR "wheat" OR "fiber" OR "fibre" OR "protein" OR "sodium" OR "salt" OR "fructose" OR "polyphenol") N3 ("free" OR "restrict*" OR "low" OR "high" OR "without" OR "eliminate*") N3 ("diet*" OR "food*" OR "eat*")) OR CI (("fat" OR "carb*" OR "calorie*" OR "gluten" OR "wheat" OR "fiber" OR "fibre" OR "protein" OR "sodium" OR "salt" OR "fructose" OR "polyphenol") N3 ("free" OR "restrict*" OR "low" OR "high" OR "without" OR "eliminate*") N3 ("diet*" OR "food*" OR "eat*"))	Search modes - Boolean/Phrase	Interface - EBSCOhost Research Databases Search Screen - Advanced Search Database - MEDLINE Complete	111,710
S13	TI (("plant-based" OR "plantbased" OR "hypocaloric" OR "hypo-caloric" OR "Mediterranean" OR DASH) N2 (diet* OR food* OR eat*)) OR AB (("plant-based" OR "plantbased" OR "hypocaloric" OR "hypo-caloric" OR "Mediterranean" OR DASH) N2 (diet* OR food* OR eat*)) OR CI (("plant-based" OR "plantbased" OR "hypocaloric" OR "hypo-caloric" OR "Mediterranean" OR DASH) N2 (diet* OR food* OR eat*))	Search modes - Boolean/Phrase	Interface - EBSCOhost Research Databases Search Screen - Advanced Search Database - MEDLINE Complete	15,163
S12	TI (vegetarian* OR vegan* OR FODMAP* OR "Dietary Approaches To Stop Hypertension" OR fruit* OR vegetable* OR "whole grain*") OR AB	Search modes - Boolean/Phrase	Interface - EBSCOhost Research Databases	216,380

	(vegetarian* OR vegan* OR FODMAP* OR "Dietary Approaches To Stop Hypertension" OR fruit* OR vegetable* OR "whole grain*") OR CI (vegetarian* OR vegan* OR FODMAP* OR "Dietary Approaches To Stop Hypertension" OR fruit* OR vegetable* OR "whole grain*")		Search Screen - Advanced Search Database - MEDLINE Complete	
S11	TI (diet* or nutrition* or food*)	Search modes - Boolean/Phrase	Interface - EBSCOhost Research Databases Search Screen - Advanced Search Database - MEDLINE Complete	486,947
S10	TI ((diet* or nutrition*) N2 (counsel* or advice or consult* or intervention or behavio* or program* or service* or assess* or therap* or individual* or personal* or habit* or recommend* or change* or improve* or education* or support* or self-manage* or management or prescription* or educat*)) OR AB ((diet* or nutrition*) N2 (counsel* or advice or consult* or intervention or behavio* or program* or service* or assess* or therap* or individual* or personal* or habit* or recommend* or change* or improve* or education* or support* or self-manage* or management or prescription* or educat*)) OR CI ((diet* or nutrition*) N2 (counsel* or advice or consult* or intervention or behavio* or program* or service* or assess* or therap* or individual* or personal* or habit* or recommend* or change* or improve* or education* or support* or self-	Search modes - Boolean/Phrase	Interface - EBSCOhost Research Databases Search Screen - Advanced Search Database - MEDLINE Complete	259,857

	manage* or management or prescription* or educat*))			
S9	TI ("dietetic*" OR "dietitian*" OR "dietician*" OR "nutritionist*" OR "nutrition worker*" OR "medical nutrition therap*") OR AB ("dietetic*" OR "dietitian*" OR "dietician*" OR "nutritionist*" OR "nutrition worker*" OR "medical nutrition therap*") OR CI ("dietetic*" OR "dietitian*" OR "dietician*" OR "nutritionist*" OR "nutrition worker*" OR "medical nutrition therap*")	Search modes - Boolean/Phrase	Interface - EBSCOhost Research Databases Search Screen - Advanced Search Database - MEDLINE Complete	26,042
S8	MH ("Micronutrients+" OR "Vitamin E+" OR "Calcium, Dietary" OR "Iron, Dietary" OR "Iron" OR "Ferritins+" OR "Zinc" OR "Vitamin B Complex" OR "Vitamin B 6+" OR "Vitamin B 12+" OR "Folic Acid+" OR "Niacin" OR "Riboflavin" OR "Magnesium")	Search modes - Boolean/Phrase	Interface - EBSCOhost Research Databases Search Screen - Advanced Search Database - MEDLINE Complete	432,180
S7	MH ("Food+" OR "Diet+")	Search modes - Boolean/Phrase	Interface - EBSCOhost Research Databases Search Screen - Advanced Search Database - MEDLINE Complete	951,828
S6	MW diet therapy	Search modes - Boolean/Phrase	Interface - EBSCOhost Research Databases Search Screen - Advanced Search	65,507

			Database - MEDLINE Complete	
S5	MH ("diet+" OR "diet therapy" OR "Vegetarians+")	Search modes - Boolean/Phrase	Interface - EBSCOhost Research Databases Search Screen - Advanced Search Database - MEDLINE Complete	339,612
S4	MH (dietetics OR nutritionists OR "nutrition therapy" OR "nutrition assessment")	Search modes - Boolean/Phrase	Interface - EBSCOhost Research Databases Search Screen - Advanced Search Database - MEDLINE Complete	29,227
S3	S1 OR S2	Search modes - Boolean/Phrase	Interface - EBSCOhost Research Databases Search Screen - Advanced Search Database - MEDLINE Complete	46,148
S2	TI (MASHD OR NAFLD OR NASH OR “metabolic dysfunction-associated steatohepatitis” OR “metabolic dysfunction-associated steatotic liver” OR “metabolic dysfunction-associated fatty liver” OR “Nonalcoholic fatty liver” OR “Non-alcoholic fatty liver” OR “nonalcoholic	Search modes - Boolean/Phrase	Interface - EBSCOhost Research Databases Search Screen - Advanced Search	46,148

	steatohepatitis” OR “non-alcoholic steatohepatitis”) OR AB (MASHD OR NAFLD OR NASH OR “metabolic dysfunction-associated steatohepatitis” OR “metabolic dysfunction-associated steatotic liver” OR “metabolic dysfunction-associated fatty liver” OR “Nonalcoholic fatty liver” OR “Non-alcoholic fatty liver” OR “nonalcoholic steatohepatitis” OR “non-alcoholic steatohepatitis”) OR CI (MASHD OR NAFLD OR NASH OR “metabolic dysfunction-associated steatohepatitis” OR “metabolic dysfunction-associated steatotic liver” OR “metabolic dysfunction-associated fatty liver” OR “Nonalcoholic fatty liver” OR “Non-alcoholic fatty liver” OR “nonalcoholic steatohepatitis” OR “non-alcoholic steatohepatitis”)		Database - MEDLINE Complete	
S1	MH (Non-alcoholic Fatty Liver Disease)	Search modes - Boolean/Phrase	Interface - EBSCOhost Research Databases Search Screen - Advanced Search Database - MEDLINE Complete	24,234
